# Assessing basic motor competences, physical fitness, and executive function in 4-5-year-old children: a longitudinal study in a primary care setting

**DOI:** 10.1186/s13052-024-01674-1

**Published:** 2024-05-31

**Authors:** Gaizka Legarra-Gorgoñon, Yesenia García-Alonso, Robinson Ramírez-Vélez, Loreto Alonso-Martínez, Mikel Izquierdo, Alicia M Alonso-Martínez

**Affiliations:** 1 Hospital Universitario de Navarra (HUN), Universidad Pública de Navarra (UPNA), Instituto de Investigación Sanitaria de Navarra (IdiSNA), Navarrabiomed, Pamplona (Navarra), España; 2 Department of Health Sciences, Universidad Pública de Navarra, Avenida de Barañain s/n, Pamplona (Navarra), 31008 Spain; 3 CIBER of Frailty and Healthy Aging (CIBERFES), Instituto de Salud Carlos III, Madrid, Spain

**Keywords:** Motor skills, Physical fitness, Children, Preschoolers, Executive function

## Abstract

**Background:**

To evaluate the progression of physical fitness (PF), basic motor competence (BMC), and executive function (EF) over one year in children aged 4–5 years at a health center.

**Methods:**

In this longitudinal analysis, children’s BMC was evaluated using the MOBAK KG test for object and self-movement. The PREFIT Battery gauged PF through handgrip strength, standing long jump, and other fitness measures, while the Early Years Toolbox appraised EF.

**Results:**

Adjustments for confounding factors showed notable improvements in BMC, particularly in object movement (OM; mean difference 0.789, *p* = 0.044) and self-movement (SM; mean difference 0.842, *p* = 0.037), with overall MOBAK scores also increasing (mean difference 1.632, *p* = 0.018). Enhancements in the standing long jump (mean difference 9.036 cm, *p* = 0.014) and EF tasks “Mr. Ant” (mean difference 0.669, *p* < 0.001) and “Go/No-Go” (mean difference 0.120, *p* < 0.001) were evident, signifying substantial BMC gains and some progress in PF and EF.

**Conclusion:**

This research underscores the positive impact of regular training on BMC and PF in young children. Significant BMC development and associated improvements in PF and EF over the study period highlight the importance of structured activities in early childhood. These findings advocate for standardized training programs to enhance childhood health and encourage active lifestyles.

**Trial registration:**

NCT05741879. Registered February 14, 2023, Version 1.

## Background

Throughout normal child development, particularly in preschool-aged children, basic motor competencies (BMC), physical fitness (PF), and executive function (EF) concurrently develop [1]. Childhood is a crucial period for developing motor skills [2], where acquiring and developing BMC is an essential component for individuals’ physical, psychological and social growth and well-being throughout life [3]. Preschool BMC have been linked to health outcomes like adiposity [4], self-stem [5], cardiorespiratory capacity [6] and cognition [7]. During early childhood, progress in body control and mental processing abilities indicate overall development [8]. Encouraging BMC development in preschool is important because BMC directly connects to physical health, academic performance, and psychosocial well-being in children [9].

The preschool stage represents a vital period in children´s motor development where acquiring and refining BMC play a crucial role in overall development [10]. From a motor development perspective, BMC establishes an important basis for subsequently learning specific skills. Preschoolers undergo swift changes in motor coordination [11]. As children grow, they show progressive refinement in motor competence from accumulating motor experiences that spur motor control development [12–14].

It has commonly been assumed that PF signifies a powerful health marker in both childhood and adulthood, encompassing cardiovascular endurance, muscular strength and speed, all of which progressively increase during child development [1, 15]. On the other hand, EF includes cognitive skills that enable logical thinking, planning, problem-solving and life management [16]. EF has been linked to improved physical and mental health and performance in academic and everyday settings [17, 18].

Previous studies examined preschoolers in educational context [19, 20]. The novelty of our study involves the assessment of BMC, PF, and EF in 4-5-Year-Olds in a primary care center. We hypothesize that the population sample will be more heterogeneous in terms of socio-economic-cultural levels compared to previous studies. Assessing motor competence in a longitudinal primary care setting is vital for capturing the evolution of these skills where children frequently receive health check-ups, representing the general population and informing public health initiatives. The longitudinal design tracks individual progress, distinguishing typical development from potential delays or disorders, particularly crucial in early years when establishing BMC is strongly linked to subsequent health. Situating research in a real-world context ensures direct applicability of findings to healthcare providers, facilitating the implementation of evidence-based practices to promote healthy motor development.

In summary, evidence indicates positive associations between physical activity (PA), PF, cognition and academic achievement [19]. Cardiovascular capacity and muscular strength develop over time, typically showing improvements with growth and maturation [20]. Therefore, the aim of this study was to assess PF, BMC and EF in 4-year-old children and analyse their development one year later at 5 years of age in a primary care setting.

## Methods

### Study design and participants

A longitudinal study, part of the “Observatorio y programa de Intervención de ejercicio físico y estilos de vida en familia para niños y niñas de 4 a 5 años en Atención Primaria” project, conducted two assessments one year apart (2022–2023) and can be explored further at observatorioactividadfisica.es. The initial cohort comprised 70 children (38 boys, 32 girls) with an average age of 4.83 (± 0.49) years. Assessments were integrated into their routine primary care over the year, with 11 children failing to complete the second measurement—3 due to a change in their primary care center and 8 absent from the follow-up assessment. Families were informed of study purposes. Children provided oral assent and legal guardians written informed consent. Participants were 4-5-year-old children from the Iturrama primary care center in Pamplona, Spain, excluding recent injuries/surgeries or physical testing limitations or heart or respiratory system problems.

The protocol aligned with the Declaration of Helsinki following Ethics Committee of the Department of Health of Navarra approval (PI_2021/111).

### Measures and procedures

The recruitment of participating families for the study was facilitated by the medical staff at the primary care centers. The personnel responsible for collecting PF data were professionals with expertise in PF and EF assessment. They received comprehensive training from the research staff of the coordinating center, the e-FIT UPNA Research Group.

Anthropometrics included height (cm), weight (kg), and body mass index (BMI) per CDC-NHANES protocols with trained assessors [21]. Height was measured in the Frankfurt position using a SECA 213® stadiometer (1-mm precision). Weight was measured with a Tanita DC-430MAS® scale (100-g precision) in light clothing without shoes. BMI was calculated as the ratio of an individual’s weight in kilograms divided by the height in meters squared (kg/m2). Waist circumference was measured to the nearest 1 mm at the umbilicus using a SECA 201 calibrated tape.

PF, encompassing cardiorespiratory fitness (CRF), lower and upper body strength, and speed-agility, was appraised using the PREFIT battery [21]. This battery is acknowledged as a practical and dependable means to evaluate physical fitness in preschool-aged children [22]. The circuit-based test was demonstrated then individually performed, except the 20 m in small 6-child subgroups. Upper limb strength was measured by hand grip with a Takei 5001® analog dynamometer squeezed for 2–3 s [23]. Children did two alternating hands with the higher value retained; their average comprised upper-body strength.

Lower limb strength was measured by the SLJ. Children performed a maximum horizontal jump from standing, landing on both feet while maintaining upright posture. Three attempts occurred with the best result (cm) recorded. The 4 × 10 m test assessed speed-agility. Children ran twice between two lines 10 m apart, covering 40 m total, with full recovery between attempts. The fastest time (seconds) was analyzed. CRF used the adapted 20 m shuttle run where children ran between lines 20 m apart. An audio signal of increasing pace (starting 6.5 km/h and elevating 0.5 km/h per minute) was used. The test ended when the child failed two consecutive attempts to reach a line or stopped due to exhaustion. Children performed one test with total laps recorded.

Standardized values (z-scores) were calculated per test by subtracting individual values from mean test values, then dividing the difference by the test standard deviation. Continuous scores were derived for each of the four selected fitness components separately for boys and girls. Higher PF z-scores indicate superior fitness.

The validated MOBAK KG test battery assessed BMC in this age group [24]. This 8-test battery measures preschooler (ages 4–6) object movement (OM) (throwing, catching, bouncing, dribbling) and self-movement (SM) (balancing, rolling, jumping, running) skills. Both subscales have maximum 8 points, producing a 0 (lowest) to 16 (highest) combined MOBAK score.

Participants performed tasks without prior attempts. “Throwing” and “catching” involved 6 attempts scoring: 0–2 attempts = 0 points; 3–4 attempts = 1 point; 5–6 attempts = 2 points. “Bouncing”, “dribbling”, “balancing”, “rolling”, “jumping” and “running” involved 2 attempts per task scored dichotomously (0 = fail, 1 = succeed), summed as: 0 points for 0 successes; 1 point for 1 success; 2 points for 2 successes. Identifying skill strengths/weaknesses informs targeted support areas.

EF assessment involved researchers administering digital iPad tests from the Early Years Toolbox TM (EYT-2017) comprising “Mr. Ant” and “Not This” for memory, “Card sorting” for cognitive flexibility, and “Go/No-Go” for inhibitory control. The Early Years Toolbox (EYT) is a collection of iPad measures assessing young children’s emerging cognitive, self-regulatory, language, numeracy, and social development through game-like assessments. EYT represents an advance over existing measures by capturing abilities shown predictive of later academic, social, emotional, cognitive and life outcomes. Researchers administered four EYT tasks: “Mr. Ant” and “Not This” for memory; “Card sorting” for cognitive flexibility; and “Go/No-Go” for inhibitory control.

### Statistical analysis

Continuous variables were expressed as mean ± standard deviation. Normality was assessed using the Kolmogorov-Smirnov test, revealing significant BMC differences between time points in OM, SM, and total MOBAK score.

A repeated measures analysis of covariance (ANCOVA) examining anthropometric, BMC, PF, and EF differences was conducted, adjusting for age and BMI. The magnitude of the effect size was interpreted using thresholds as suggested by Cohen [43]: 0.0 to 0.19—trivial; 0.20 to 0.49—small; 0.50 to 0.79—moderate; >0.80—large. Analyses used IBM SPSS Statistics 26 with statistical significance set at *p* < 0.05.

## Results

Table 1 presents descriptive characteristic summaries for the sample’s boys and girls, including anthropometric measurements, PF, BMC, and EF. No significant gender differences emerged. Table 2 shows PF, BMC, and EF differences between the two assessments one year apart. In year two, results improved significantly— SLJ (mean difference 9.036 cm, *p* = 0.014; d = 0.439); BMC OM (mean difference 0.789 points, *p* = 0.044; d = 0.353) and SM (mean difference 0.842 points, *p* = 0.037; d = 0.388); MOBAK total score (mean difference 1.632 points, *p* = 0.018; d = 0.418); and EF “Mr. Ant” (mean difference 0.669, p = < 0.001; d = 0.868) and “Go/No-Go” (mean difference 0.120, p = < 0.001; d = 0.667).

**Table 1:** Characteristics of the study population

	4 years			5 years		
	Boys (*n* = 38)	Girls (*n* = 32)	*P*-value	Boys (*n* = 31)	Girls (*n* = 28)	*P*-value
Age	4.87(0.49)	4.77(0.49)	0.407	5.85(0.49)	5.74(0.46)	0.578
Height (cm)	108.9(4.92)	106.7(4.95)	0.070	113.93(5.22)	111.39(5.08)	0.068
Weight (kg)	19.80(3.83)	18.59(2.97)	0.150	21.04(3.10)	19.94(3.42)	0.208
Body Mass Index (kg/m2)	16.62(2.36)	16.25(1.53)	0.448	16.09(1.26)	16.09(1.65)	0.991
Waist circumference (cm)	54.9(6.18)	53.5(3.16)	0.240	55.12(3.67)	54.19(4.07)	0.371
Waist-to-height ratio	0.51(0.05)	0.50(0.03)	0.776	0.48(0.03)	0.49(0.03)	0.710

**Table 2:** Comparison of anthropometric parameters, physical fitness, basic motor competences and executive function between 4 and 5-year-old children (one-year difference)

	4 years			5 years				
		95% CI		95% CI				
	Mean	Lower	Upper	Mean difference	Lower	Upper	d	p
**Anthropometric parameters**								
Height (cm)	107.90	106.70	109.09	5.393	4.870	5.916	2.791	< 0.001
Weigh (kg)	19.25	18.42	20.08	1.756	1.505	2.008	1.125	< 0.001
Body Mass Index (kg/m^2^)	16.45	15.97	16.93	-0.159	-0.377	0.058	-0.114	0.148
Waist circumference (cm)	54.3	53.11	55.52	0.904	0.294	1.513	0.368	0.004
Waist-to-height ratio	0.50	0.49	0.51	-0.016	-0.022	-0.009	-0.702	< 0.001
**Physical fitness**								
Handgrip strenght (kg)	8.22	7.75	8.69	-0.118	-0.473	0.237	-0.068	0.507
Standing long jump (cm)	87.09	81.61	92.57	9.036	1.889	16.184	0.439	0.014
Speed/Agility 4 × 10 m (s)	15.27	14.88	15.66	-0.209	-0.737	0.278	-0.163	0.368
Cardiorespiratory fitness (laps)	29.73	26.88	32.58	-1.531	-5.764	2.703	-0.145	0.470
Physical fitness (z-score)	0.11	-0.60	0.82	0.083	-0.679	0.846	0.045	0.826
**Motor skills**								
Object movement subscale (8 points)	2.51	1.96	3.07	0.789	0.0.24	1.555	0.353	0.044
Self movement subscale (8 points)	3.21	2.35	4.07	0.842	0.054	1.630	0.388	0.037
MOBAK KG sum score	5.79	4.81	6.77	1.632	0.293	2.970	0.418	0.018
**Executive function**								
“Mr. Ant”	1.77	1.59	1.95	0.669	0.369	0.970	0.868	< 0.001
“Go/No-Go”	0.64	0.59	0.70	0.120	0.054	0.187	0.667	< 0.001
“Not This”	2.37	2.24	2.50	0.188	-0.030	0.407	0.314	0.090
“Card sorting”	7.91	7.09	8.74	0.500	-0.663	1.663	0.162	0.390

Values have been reported as mean and standard deviation

**Fig. 1:**
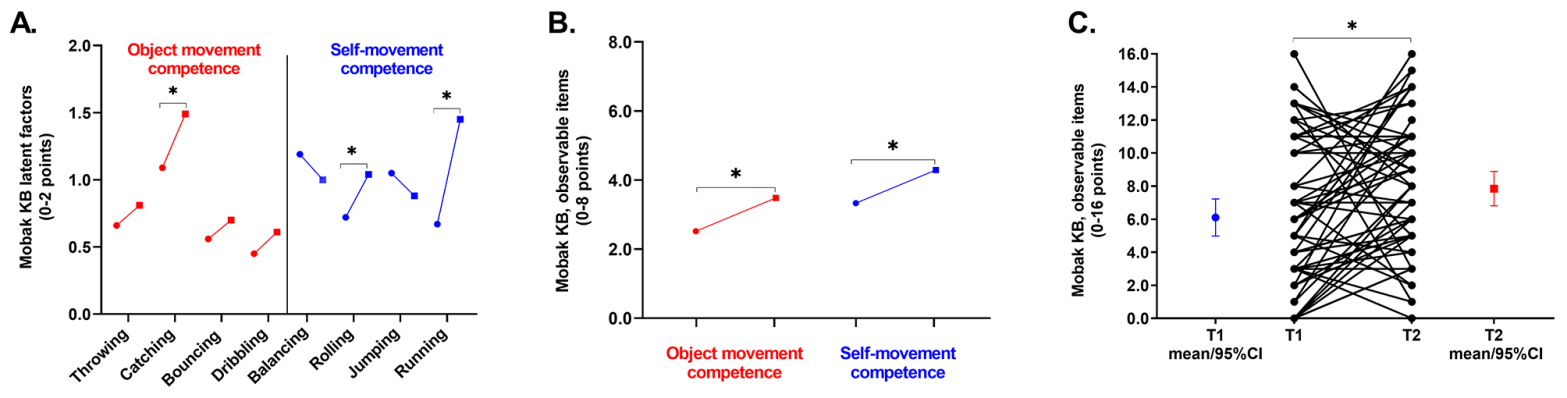
Changes in Basic Motor Competences measured through the MOBAK KG battery. Time 1 (●), Time 2 (■)

Figure 1 shows MOBAK results across the two assessments. In OM (Figure A), “catching” significantly improved (*p* = 0.006). In SM, “rolling” (*p* = 0.038) and “running” (*p* < 0.001) significantly improved. Figures B and C show significant improvements in OM (*p* = 0.044), SM (*p* = 0.037), and total score (*p* = 0.018).

## Discussion

The objective of this longitudinal study was to compare the one-year progression of PF, BMC and EF between boys and girls in a primary care setting. The main finding is the significant improvement in BMC and partial improvement in PF and EF. This study is significant due to the limited research evaluating BMC in children of these ages and due to the fact that the sample is obtained from a primary care center.

Overall, the one-year results were better than the baseline assessments. As literature shows, preschool-aged children constantly evolve motor, physical, and psychological capacities during development [25]. Developing BMC during childhood is essential for actively and successfully engaging in physical activities throughout life [26, 27]. An enhancement in proficiency of these skills at an early age could amplify the child´s perception of competence, giving to them the confidence to engage in sports later in life, thereby instigating a positive spiral of involvement from an early age [3].

Previous studies show boys tend to score higher in OM skills while girls demonstrate better balance and stability [28–30]. Our results align with this trend; boys achieved notably better BMC related to OM. These findings reflect those of Webster et al. showing superior OM performance in boys [31]. In SM skills, despite slightly higher girl scores, no significant differences emerged between boys and girls. One explanation may be the young age of our study children.

Due to typical growth and maturation, children´s BMC will naturally improve even in the absence of targeted interventions [32]. Nevertheless, the score obtained after one year of follow-up is close to the mean value of the MOBAK KG sum score. This indicates that our study population was below average (< 8 out of 16 points) initially, and after a year, despite statistically significant improvements, they still do not reach half of the total score.

We hypothesize that these low values may be attributed to low levels of PA. Several studies have documented the positive outcomes associated with PA [33]. For this reason, it is crucial not to underestimate the importance of adhering to the PA recommendations established by the World Health Organization [33].

We also observed improved EF, particularly on the “Mr. Ant” memory and Go/No-Go inhibitory control tests. These results support evidence that motor and cognitive development interconnect through common processes like sequencing, monitoring, and planning [34]. They also align with previous observations of a positive BMC and EF improvement association [35].

Regarding PF results, we observed significant SLJ test improvement. However, HGS and 20 m shuttle run PREFIT outcomes slightly decreased at the second measurement. These surprising findings contradict literature suggesting childhood PF improves over time [36]. Increased screen time and physical inactivity offer one explanation [37]. Still, interpret these results cautiously given our non-representative sample. Considering all results, we can hypothesize BMC acquisition precedes PF, as BMC improved more easily than PF. This underscores the importance of developing BMC to improve PF.

Existing literature has delineated the correlation between PF and BMC [28, 38, 39]. Studies focusing on BMC have consistently reported positive outcomes from a variety of training programs, ranging in duration from 2 to 10 months, with frequencies of 2–3 times per week, conducted in school or home settings [40]. A unique finding of these interventions is the successful enhancement of BMC among preschool-aged children. The review by Jones et al. further substantiates the value of reinforcing these foundational abilities, highlighting a beneficial link between them and early years physical activity (PA) [41]. Therefore, the promotion of BMC development and practice warrants more definitive guidelines and specialized training for educators. Future initiatives should aim to emphasize BMC skills training as a contribution to the bolstering of overall childhood PF and health. Given that BMC development is pivotal and consolidates during the early stages of life, laying this foundation is essential for encouraging ongoing engagement in PA [42].

A major strength of this study was using the MOBAK test battery to measure BMC in 4-5-year-olds an innovative approach given the dearth of BMC evidence in this population. Additionally, these findings are particularly remarkable because the recruitment was from a primary care center. The PREFIT tests deserve mention for their reliability and validity in assessing childhood PF.

Despite strengths, the one-year longitudinal design limits observations. Tracking children over several years would better elucidate development. Additionally, our non-representative sample size from one primary care center reduces generalizability. For comparable projects in the future, it is advisable to implement strategies aimed at minimizing the number of participants who drop out between assessments. Finally, Spanish BMC reference values for MOBAK tests in this age group are unavailable, globally complicating result comparisons. Diverse assessment tools and no measurement consensus highlight the need for an internationally standardized motor competence tool, an interesting research direction.

## Conclusions

In conclusion, this one-year longitudinal study adds valuable BMC evidence in an under-researched population. Our results align with literature on BMC differences between boys and girls, as well as connections between developing BMC and EF. Partial PF improvements warrant further investigation given contradictory results. Standardizing BMC assessment and implementing early skills training offer important future research and practical directions for supporting childhood health.

## Data Availability

The datasets used and/or analyzed during the current study can be made available from the corresponding author on reasonable request.

## Abbreviations

PA: Physical activity
BMC: Basic Motor Competences
BMI: Body mass index
CRF: Cardiorespiratory fitness
EF: Executive Function
HGS: Handgrip strength
KGF: Kilograms of Force
MOBAK KG: Motorsiche Basiskompetenzen Kindergarden
OM: Object movement
PF: Physical fitness
SLJ: Standing long jump
SM: Physical activity
